# Feminization of pheromone-sensing neurons affects mating decisions in *Drosophila* males

**DOI:** 10.1242/bio.20147369

**Published:** 2014-01-17

**Authors:** Beika Lu, Kathleen M. Zelle, Raya Seltzer, Abraham Hefetz, Yehuda Ben-Shahar

**Affiliations:** 1 Department of Biology, Washington University in St Louis, MO 63130, USA; 2 Department of Zoology, George S. Wise Faculty of Life Sciences, Tel Aviv University, 69978 Tel Aviv, Israel

**Keywords:** Fruit fly, Courtship, *ppk23*, *Poxn*, *transformer*, DEG/ENaC

## Abstract

The response of individual animals to mating signals depends on the sexual identity of the individual and the genetics of the mating targets, which represent the mating social context (social environment). However, how social signals are sensed and integrated during mating decisions remains a mystery. One of the models for understanding mating behaviors in molecular and cellular terms is the male courtship ritual in the fruit fly (*Drosophila melanogaster*). We have recently shown that a subset of gustatory receptor neurons (GRNs) that are enriched in the male appendages and express the ion channel *ppk23* play a major role in the initiation and maintenance of male courtship via the perception of cuticular contact pheromones, and are likely to represent the main chemosensory pathway that influences mating decisions by males. Here we show that genetic feminization of *ppk23*-expressing GRNs in male flies resulted in a significant increase in male–male sexual attraction without an apparent impact on sexual attraction to females. Furthermore, we show that this increase in male–male sexual attraction is sensory specific, which can be modulated by variable social contexts. Finally, we show that feminization of *ppk23*-expressing sensory neurons lead to major transcriptional shifts, which may explain the altered interpretation of the social environment by feminized males. Together, these data indicate that the sexual cellular identity of pheromone sensing GRNs plays a major role in how individual flies interpret their social environment in the context of mating decisions.

## Introduction

Sexually reproducing animals often show sexually dimorphic behaviors. One of the best-characterized models for understanding the role of genetics and neural circuits in controlling sex-specific behaviors is the fruit fly *Drosophila melanogaster* (Anand et al., 2001; Demir and Dickson, 2005; Manoli et al., 2005; Rideout et al., 2010; Ryner et al., 1996; Siwicki and Kravitz, 2009; Villella et al., 1997; Villella and Hall, 2008). Several studies have indicated that sex-specific innate mating behaviors are determined by a dedicated neuronal circuit that comprises neurons in the central and peripheral systems, and of which development and function are determined by the sex-specific splicing of the transcription factors *fruitless* (*fru*) and *doublesex* (*dsx*) (Manoli et al., 2005; Rideout et al., 2010; Stockinger et al., 2005).

Cuticular hydrocarbons (CHCs) serve as contact sex pheromones in flies and other insects (Ferveur, 2005; Kent et al., 2008; Krupp et al., 2008; Kuo et al., 2012; Yew et al., 2009). These data suggest that the gustatory system is likely to play an important role in the detection of sex-specific stimuli. This is supported by findings that several members of the gustatory receptor family play a role in the detection of pheromonal signals (Bray and Amrein, 2003; Koganezawa et al., 2010; Miyamoto and Amrein, 2008; Moon et al., 2009; Wang and Anderson, 2010; Wang et al., 2011; Watanabe et al., 2011). In addition, we and others have recently shown that a subset of sexually dimorphic GRNs in the male and female forelegs express both *fru* and the ion channel *ppk23*, and are likely the primary contact pheromone sensory neurons in the adult fly (Lu et al., 2012; Thistle et al., 2012; Toda et al., 2012). Because *ppk23* seems to be exclusively expressed in *fru*-positive gustatory sensory neurons in the male appendages but not in any *fru*-positive central neurons (Lu et al., 2012), studies of the effects of these neurons on male courtship behavior represent an excellent opportunity to study the relative contribution of the gustatory system to courtship decisions, independent of the brain.

Although stereotypic, both the perception and production of pheromones is highly plastic across sex, species, and physical and social environmental conditions (Billeter et al., 2012; Everaerts et al., 2010; Ferveur, 2005; Kent et al., 2008; Krupp et al., 2008). Here we show that feminization of *ppk23/fru*-specific GRNs in the male appendages is sufficient to mimic the effects of mutations in the *fru* locus on male sexual behaviors, independent of the role of *fru*^M^ in the brain. Our data suggest a simple behavioral model in which *ppk23*-expressing GRNs represent a focal integration point of social environmental cues and the genetic factors that determine cellular sexual identity, which together influence mating decisions of males.

## Results

### Feminization of *ppk23*-expressing GRNs induces male–male courtship without altering the innate sexual preference for females

In previous work we have shown that the ion channel *ppk23* and the gustatory neurons that express it play an essential role in the initiation and maintenance of normal male courtship behavior (Lu et al., 2012), by demonstrating that both mutations in *ppk23* and blocking the activity of *ppk23*-expressing GRNs led to a defective male–female courtship behavior. On the other hand, we did not observe any effects of these manipulations on male–male courtship (Lu et al., 2012). We interpreted these data as suggesting that *ppk23*-expressing GRNs were mediating the behavioral response of males to aphrodisiac CHCs, which was further confirmed by the reduced behavioral response of *ppk23* mutant males to the excitatory pheromone 7,11-heptacosadiene (7,11 HD) (Lu et al., 2012; Thistle et al., 2012). However, a calcium imaging study suggested that at least some *ppk23*-expressing GRNs can also respond to the inhibitory pheromone 7-tricosene (7-T) (Thistle et al., 2012). Together, these data suggested that *ppk23*-expressing GRNs represent a heterogeneous population of gustatory-like sensory neurons that are tuned to various classes of contact pheromones.

**Fig. 1.**
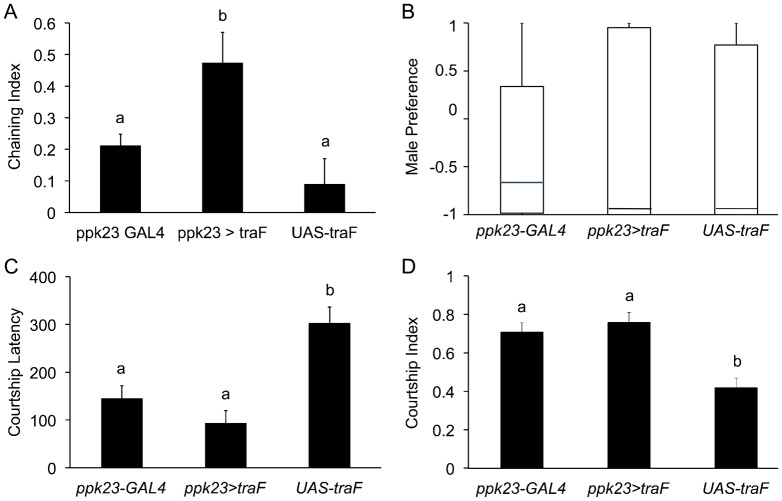
Males with feminized *ppk23*-expressing GRNs show increased male–male courtship behavior. (A) Male–male chaining index in feminized flies (*ppk23*>*tra*^F^) and two parental controls (*ppk23*-GAL4, UAS-*tra*^F^). Feminized males showed higher male chaining behavior relative to males from parental lines. (B) Feminized males preferred females to males in choice courtship assays. Boxplots show the distribution of choice behaviors (1, male; −1 female). (C,D) Feminized males show normal courtship behavior towards wild-type females. Feminization of *ppk23*-expressing GRNs had no effect on either latency or courtship index relative to parental *ppk23*-GAL4 males. UAS-*tra*^F^ parental males showed consistent longer latency (C) and reduced courtship index (D), which were likely due to unrelated factors present in the genetic background of this specific transgenic line. The different letters (a,b) in parts A, C, and D represent groups that are significantly different from each other based on ANOVA *post hoc* tests.

*ppk23*-expressing GRNs in the forelegs are sexually dimorphic, and express post-mitotically the sex-determination transcription factor *fruitless* (*fru*) (Lu et al., 2012; Thistle et al., 2012; Toda et al., 2012). Sex-determination factors such as *fru* and *dsx* are spliced into male or female-specific transcripts by the sex-specific splicing factor *transformer* (*tra*). Previous studies showed that overexpression of the female-specific transcript of *tra* (*tra*^F^) is sufficient to induce female-like differentiation in male tissues, including the nervous system (Ferveur et al., 1997; Ferveur et al., 1995). Consequently, we hypothesized that feminization of *ppk23*-expressing GRNs with ectopic expression of *tra*^F^ in otherwise intact males will disrupt their normal function and will lead to similar mating phenotypes we observed in *ppk23* mutant males. To our surprise, males with feminized *ppk23*-expressing GRNs showed robust male–male courtship behaviors measured by male chaining behavior (ANOVA, *n* = 6–8 groups, *p*<0.01**) (Fig. 1A). However, *ppk23*-feminized males retained their overall sexual preference for courting females when given a choice between wild-type male and female targets (Kruskal–Wallis rank sum test, *p* = 0.39) (Fig. 1B), and showed an overall normal courtship behavior towards wild-type females measured by courtship latency and index (Fig. 1C,D). These observations were in stark contrast to the inhibition of male courtship that we previously observed when *ppk23*-expressing cells were blocked by the ectopic expression of the tetanus toxin in these cells (Lu et al., 2012).

We originally identified *ppk23* as a gustatory-enriched Degenerin/epithelial sodium channel (DEG/ENaC) by screening for genes that were not expressed in the *Poxn* mutant (Lu et al., 2012). Animals that carry mutations in *Poxn* lack all external gustatory sensilla (Awasaki and Kimura, 1997; Boll and Noll, 2002; Dambly-Chaudière et al., 1992; Nottebohm et al., 1992; Nottebohm et al., 1994; Vervoort et al., 1995). *Poxn* also retains its expression in all postmitotic GRNs and thus serves as an excellent marker for these neurons. As a result, we hypothesized that if the effects of feminizing *ppk23*-expressing GRNs are indeed due to gustatory functions, then feminizing the complete gustatory sensory system in males should lead to a phenotype that is similar to the one we observed in *ppk23*-feminized males. To completely feminize the gustatory system we expressed UAS-*tra*^F^ with a previously published *Poxn*-GAL4 line (Boll and Noll, 2002). As we expected, males with feminized GRNs showed a robust chaining behavior that was indistinguishable from males with the feminization of *ppk23*-expressing GRNs only (supplementary material Fig. S1A). However, in contrast to *ppk23*-feminized, *Poxn*-feminized males showed a clear preference to courting males over females (supplementary material Fig. S1B). Nevertheless, when offered a wild-type female as a mating target, *Poxn*-feminized males actively courted virgin females with the same tenacity as parental and sibling controls (supplementary material Fig. S1C,D). These data indicated that courtship decisions in males were also affected by *ppk23*-independent GRNs, and suggested that ectopic feminization of the gustatory sensory system was sufficient to induce a dramatic shift from heterosexual to homosexual behaviors in *Drosophila* males. In both *ppk23*-GAL4 and *Poxn*-GAL4 studies we used the parental lines as wild-type controls as has been described in previous studies that used the UAS-*tra*^F^ transgene (Fernández et al., 2010; Hoxha et al., 2013; Lazareva et al., 2007; Shirangi et al., 2013). Although our data suggest that the homozygous UAS-*tra*^F^ parental line shows some male chaining behavior, our analyses indicated that chaining is significantly higher when *tra*F was expressed by either *ppk23*-GAL4 or *Poxn*-GAL4. Thus, we conclude that feminization of chemosensory neurons was sufficient to induce chaining behavior in males.

### Feminization of ppk23-expressing cells does not increase the sexual attractiveness of manipulated males

Although we did not observe expression of *ppk23* outside the chemosensory system, it is still possible that some of the observed effects on male–male courtship were due to qualitative or quantitative changes in the production of cuticular pheromone signals in feminized males via direct or indirect effects on the pheromone producing oenocytes (Billeter et al., 2009). To test this possibility we first examined the attractiveness of *ppk23*-feminized males as courtship targets for wild-type males. We expected that wild-type males would become more sexually attracted to feminized males than non-feminized males. However, our data indicated that the attractiveness of manipulated males did not differ from wild-type parental controls (supplementary material Fig. S2A,B). We also analyzed the CHC profiles of feminized and wild-type parental males by using gas chromatography (FID) and combined gas chromatography/mass spectrometry (GC/MS). As with behavior, we did not observe a significant effect of the *ppk23*-feminization on the overall CHC profile or any of the individual compounds (supplementary material Fig. S2C). These data indicate that the observed increase in male–male courtship in feminized males is due to changes in sensory functions rather than their pheromonal signature.

### Feminization of GRNs does not alter gross axonal wiring patterns in the thoracic ganglion

**Fig. 2.**
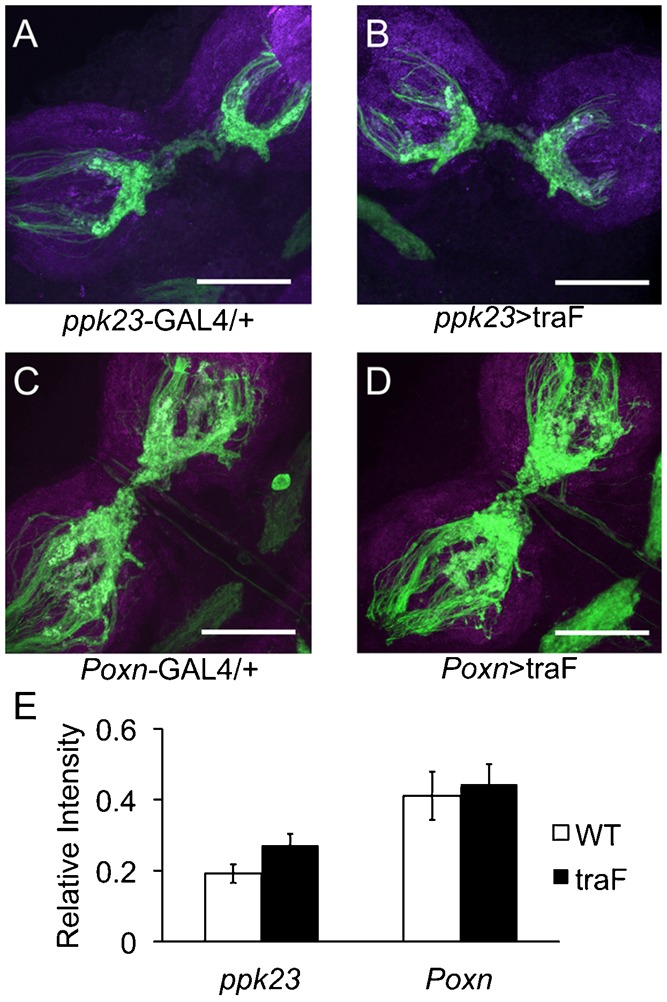
Feminization of *ppk23*-expressing chemosensory neurons does not affect their gross axonal projection patterns. (A) Membrane-tethered GFP (UAS-mCD8::GFP) was expressed by *ppk23*-GAL4 (wild-type pattern). (B) UAS-mCD8::GFP was co-expressed with UAS-*tra*^F^ by *ppk23*-GAL4. (C) mCD8::GFP was expressed by the pan-gustatory *Poxn*-GAL4 line. (D) UAS-mCD8::GFP was co-expressed with UAS-*tra*^F^ by *Poxn*-GAL4. (E) Quantification of relative fluorescence intensity in the midline-crossing region. No significant differences were found between control and *tra*^F^-expressing males with either GAL4 lines. Scale bars: 25µm.

*ppk23*-expressing GRNs are about two-fold more abundant in male relative to female forelegs, and show a sexual dimorphic axonal midline crossing in the thoracic ganglia of males but not females (Lu et al., 2012). It has been shown that the axonal midline crossing of GRNs in the male depends on the expression of the male forms of the two main sex-determination transcription factors *fru* and *dsx* (Mellert et al., 2010). Since the splicing of both *fru*^M^ and *dsx*^M^ depends on the sex-dependent splicing of *tra* (Robinett et al., 2010; Verhulst et al., 2010), we hypothesized that the ectopic expression of *tra*^F^ in *ppk23*- or *Poxn*-expressing GRNs in males may have resulted in the inhibition of axonal midline crossing, which subsequently led to aberrant male sexual behaviors. However, anatomical analyses of midline crossing in feminized *ppk23* or *Poxn* males revealed no gross changes in axonal wiring patterns relative to wild-type controls (Independent sample t-tests; *n* = 5–6 per genotype) (Fig. 2A–E). We also did not observe any effects of feminization of overall number of *ppk23*-positive cells in males or females (supplementary material Fig. S3). We cannot explain why feminization by the ectopic expression of *tra*^F^ did not inhibit axonal midline crossing as was previously reported for direct manipulations of the *fru*^M^ transcripts in *Poxn* neurons (Mellert et al., 2010). Nevertheless, our data suggest that the behavioral outcomes of chemosensory feminization are not directly related to the status of axonal midline crossing or to the relative abundance of *ppk23*-positive cells in forelegs.

The simplest possible explanation for our findings is that feminization of *ppk23*-expressing GRNs lead to increased male chaining behavior was due to their reduced detection of a inhibitory signals from other males but without affecting their response to excitatory signals from females. To test this hypothesis we examined the behavioral response of males to the inhibitory pheromone 7-T, which is sufficient to inhibit male–male courtship (Billeter et al., 2009; Fernández et al., 2010; Ferveur and Sureau, 1996; Krupp et al., 2008). Therefore, we examined the effect of feminization of *ppk23*-expressing GRNs on the behavioral response of manipulated and control males to 7-T. Our data show that in contrast to our hypothesis, feminized males were still sensitive to the inhibitory effects of 7-T when responding to perfumed decoys (supplementary material Fig. S4A,B). These data suggested that the increase in male–male courtship behavior was not due to a reduced sensing of the principle inhibitory pheromone 7-T, and may suggest that feminized males are actively attracted to other males due to ectopic changes in chemosensory functions.

**Fig. 3.**
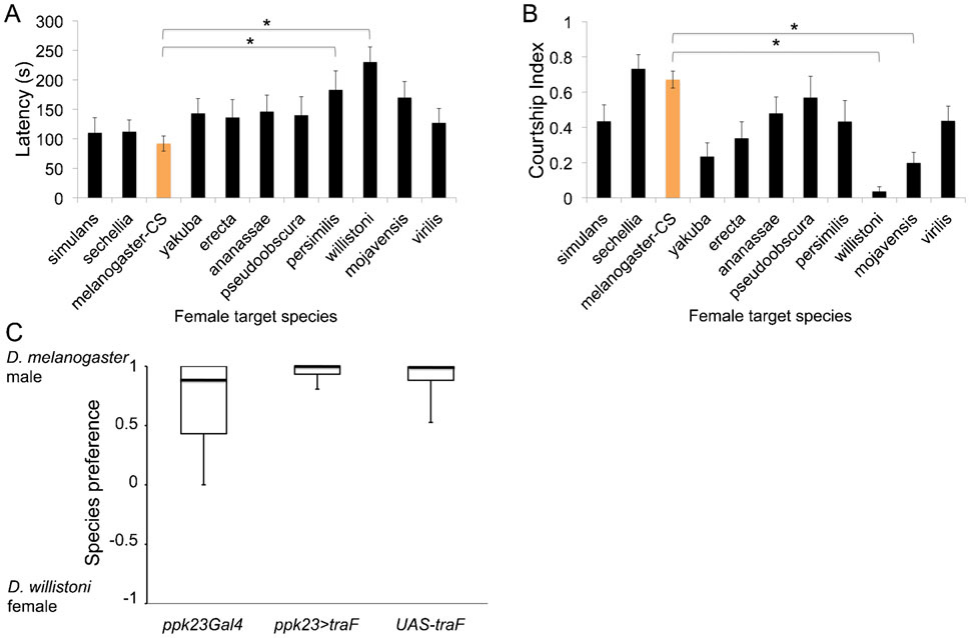
*D. melanogaster* males prefer to court conspecific males over females of a distant species. Wild-type *D. melanogaster* males courted females from other species with varying degrees of intensity as measured by the courtship latency (A) and the courtship index (B). (C) In choice assays, *D. melanogaster* males of all tested genotypes chose to court conspecific males over females of *D. willistoni*. Boxplots represent the distribution of male mating choices. No significant differences were found between feminized flies and the parental controls. *p<0.05.

Our data indicate that males with feminized *ppk23*-expressing cells court conspecific males, but when given a choice between the sexes, still prefer to court conspecifc females. Thus, these data could not resolve whether courting wild-types males by *ppk23*-feminized males is an active choice or whether these males will court any possible target in the absence of females. To better distinguish between these two possible explanations we next provided *D. melanogaster* wild-type males with heterospecific females from diverse *Drosophila* species of varying phylogenetic distances. Our data indicated that wild-type *D. melanogaster* males promiscuously courted most single female targets, independent of phylogenetic distances [ANOVA, *n* = 15–20 for each species except *D. melanogaster* (*n* = 61), *p<0.05] (Fig. 3A,B). However, females from *D. persimilis*, *D. willistoni* as measured by courtship latency, and *D. willistoni* and *D. mojavensis* as measured by courtship index, were significantly less attractive than other species. As a result, we hypothesized that if *ppk23*-feminized males court other *D. melanogaster* males because they actively find them attractive then when presented with a choice between a *D. melanogaster* male and an unattractive female from a different species then they will still court conspecfic males. Alternatively, if in the absence of *D. melanogaster* female, *ppk23*-femized males will court any targets without discrimination then they should court both targets equally. To test this we asked *ppk23*-feminized males to choose between between a *D. melanogaster* male and the unattractive *D. willistoni* female. To our surprise, both feminized and wild-type control males preferred *D. melanogaster* males to *D. willistoni* females as courtship targets (Kruskal–Wallis rank sum test, *n* = 10–15, *p* = 0.44) (Fig. 3C). These data further supported a model in which male sexual preferences are strongly affected by the available pool of mating targets, and that the decision to court a specific target depends on its relative attractiveness to other possible targets. Furthermore, our data indicate that the feminization of *ppk23*-expressing GRNs leads to an active choice of males as possible targets by shifting how males interpret their social environment when making courtship decisions in complex social environments.

### Feminization of ppk23-expressing GRNs leads to changes in the sensory transcriptome in the male appendages

**Fig. 4.**
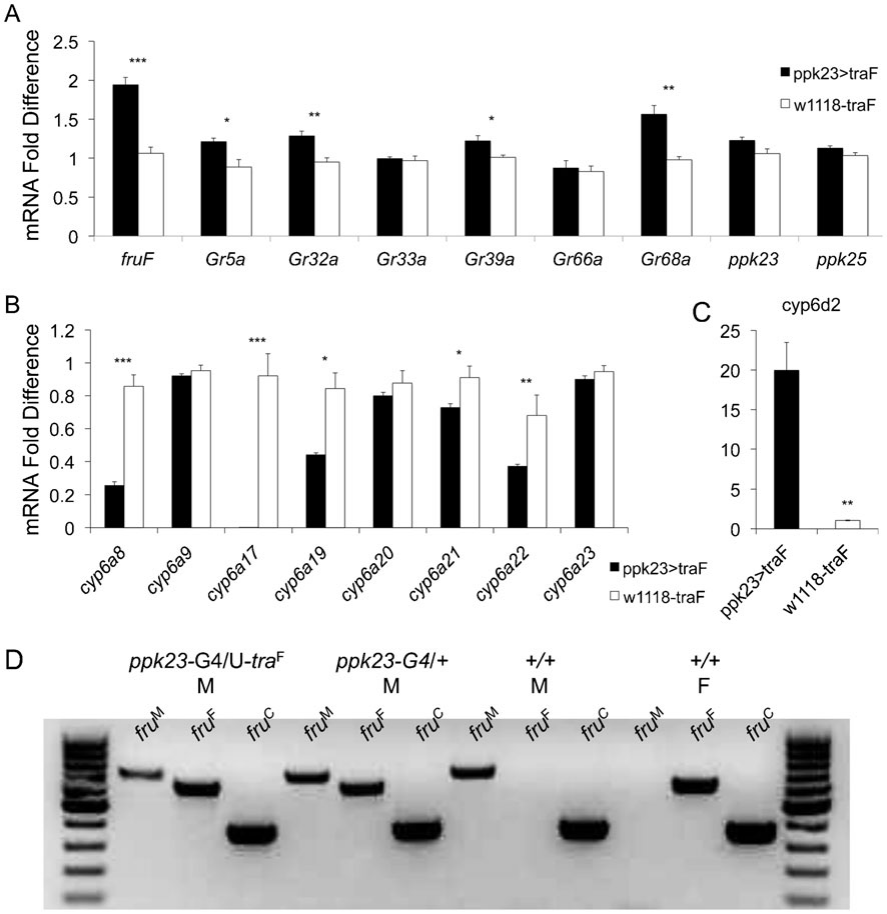
Feminization of *ppk23*-expressing cells leads to significant shifts in the chemosensory transcriptome in male appendages. (A) Real-time quantitative RT-PCR analyses of chemosensory genes that have been previously implicated in pheromonal sensing. Analyses were of total RNA extracted from male appendages from feminized flies (*w*^1118^;*ppk23-GAL4*/UAS-*tra*^F^) and wild-type controls (*w*^1118^/UAS-*tra*^F^). (B) Real-time quantitative RT-PCR analyses of members of the Cytochrome P450 family, subfamily 6. The expression of *Cyp6d2* is shown separately since this gene was regulated in the opposite direction relative to all other *Cyp6* genes (C). (D) PCR analyses of sex-specific *fru* transcripts in appendages. *fru*^M^, male-specific; *fru*^F^, female-specific; *fru*^C^, common exons. M  =  male, F  =  female, +/+ = *w^1118^*. *p<0.05; **p<0.01.

Feminization of *ppk23*-expressing GRNs did not affect the overall cell number in the forelegs of males and females (supplementary material Fig. S3), or their axonal projection patterns (Fig. 2). Therefore, we hypothesized that an alternative explanation for the observed effects of feminization on male behavior were transcriptional changes in *ppk23*-expressing GRNs. To test this hypothesis we used real-time quantitative RT-PCR to study changes in the expression of *fru^F^* and candidate genes in the male appendages in response to ectopic feminization. We focused our analysis on several genes from the *Gr* and *ppk* families, which have been previously implicated in mediating the gustatory response to contact pheromones (Ben-Shahar et al., 2010; Ben-Shahar et al., 2007; Bray and Amrein, 2003; Lin et al., 2005; Liu et al., 2012; Lu et al., 2012; Miyamoto and Amrein, 2008; Moon et al., 2009; Starostina et al., 2012; Thistle et al., 2012; Toda et al., 2012; Watanabe et al., 2011), as well genes that encode for feeding related sweet and bitter receptors (*Gr5a* and *Gr66a*, respectively) (Dahanukar et al., 2001; Marella et al., 2006; Moon et al., 2006). Although we observed statistically significant changes in the expression levels of several members of the *Gr* and *ppk* families, none of the studied receptor genes showed a dramatic change that may explain the robust behavioral outcome of *ppk23*-feminization (Fig. 4A) (Independent sample t-test; *n* = 4 for each bar; *p<0.05; **p<0.01; ***p<0.001). Furthermore, although we have previously shown that *ppk23*-expressing GRNs do not overlap anatomically with either sweet (*Gr5a*-expressing GRNs) or bitter (*Gr66a*-expressing GRNs) (Lu et al., 2012), we observed a small but significant increase in *Gr5a* expression in the appendages of feminized males relative to wild-type controls (Fig. 4A). Feminized males also showed a significant increase in their sensory sensitivity to sugar (supplementary material Fig. S5), suggesting that feminization of one GRN type may have indirectly affected the physiology of other feeding related GRNs.

The perception of pheromones by the chemosensory system also depends on rapid enzymatic removal of the perceived chemicals (Feyereisen, 2006; Oakeshott et al., 2010; Wang et al., 2008). In support of this, a gene encoding for a cytochrome P450 enzyme (*Cyp6a20*) was recently implicated in chemosensory functions underlying male–male interactions in *Drosophila* (Wang et al., 2008). Although the exact role of these enzymes in chemosensory biology is not fully understood, it is likely that secreted members of the family play a role in the breakdown of cuticular contact pheromones once they enter the lumen of chemosensory sensillum (Feyereisen, 2006; Willingham and Keil, 2004), where they possibly play a role in the removal or modifications of the sensed pheromones. However, we did not find that *Cyp6a20* was significantly regulated by the feminization of *ppk23*-expressing cells (Fig. 4B) (Independent sample t-test; *n* = 4 for each bar; *p<0.05; **p<0.01; ***p<0.001). Nonetheless, several other related family members that cluster in the same genomic region as *Cyp6a20* showed dramatic changes in their expression levels in male appendages in response to feminization, with the most dramatic patterns shown by *Cyp6a17* (Fig. 4B) and *Cyp6d2* (Fig. 4C) (Independent sample t-test; *n* = 4 for each bar; *p<0.05; **p<0.01; ***p<0.001). Thus, the expression of *tra*^F^ in *ppk23*-expressing sensory neurons in males has likely led to major qualitative and quantitative changes in the expression patterns of chemosensory receptors and other genes associated with contact pheromonal signal transduction pathways.

Unexpectedly, we found that the expression of *fru*^F^ in the appendages of *ppk23*-feminized males was only about 2-fold higher than in our control line (Fig. 4A). Since our control flies included one copy of the UAS-*tra*^F^ transgene, these data suggested that this UAS line might be expressing some levels of *tra*^F^ even when GAL4 is not present. To test this, we used PCR to amplify male-specific, female-specific, and common *fru* exons in control and *ppk23*-feminized males, as well as wild-type males and females as positive controls. We found that males carrying one copy of the UAS-*tra*^F^ transgene expressed *fru*^M^ and *fru*^F^ (Fig. 4D), indicating partial level of feminization, which is likely due to a “leaky” UAS transgene.

## Discussion

Courtship in *Drosophila melanogaster* is one of the best-characterized animal mating behaviors at the molecular and cellular levels (Villella and Hall, 2008). However, we still know relatively little about how flies sense and integrate sex-specific sensory signals (Dickson, 2008). Previous studies of one of the primary sex-determination factors *fru* indicated that mutations in this gene lead to male chaining behavior (Anand et al., 2001; Demir and Dickson, 2005; Gailey and Hall, 1989; Goodwin et al., 2000; Lee et al., 2000; Manoli et al., 2005; Ryner et al., 1996). In this study we show that genetic feminization of the contact pheromone chemosensory neurons in the male fruit fly appendages is sufficient to phenocopy the classic *fru* behavioral male chaining phenotype (Fig. 1A). However, in contrast to *fru* mutant males who do not discriminate between males and females (Villella et al., 1997), *ppk23*-feminized males still retained their overall preference for courting females over males (Fig. 1B). Thus, our studies indicate that the behavioral impact of feminizing pheromone-sensing neurons on male courtship behavior cannot be explained solely by changes in *fru*-dependent processes. Nevertheless, our data clearly demonstrate that qualitative changes in the expression of chemosensory-related genes are associated with sensory feminization, suggesting that the transcription of some molecular sensory receptors is under the influence of the sex-determination pathway, and may explain some of the differences in pheromone driven behaviors in males and females (Fig. 4).

Previous studies indicated that the decision of a male to court a specific target is mediated by both attractive and repulsive signals (Billeter et al., 2009; Fernández et al., 2010; Kent et al., 2008; Krupp et al., 2008), and it is the summation of these two opposing forces that determines the length of courtship latency and the intensity of the courtship behavior once a male is committed to a specific target (Ferveur and Sureau, 1996). We found that males with feminized *ppk23*-expressing sensory neurons courted other males, but when given a choice between a male or a female *D. melanogaster* they still preferred to court a female (Fig. 1). These data indicate that feminization did not abolish the ability of these males to discriminate between males and females but rather reduced the inhibition of male–male attraction. A previous study indicated that wild-type males find animals that do not produce any cuticular hydrocarbons, and hence do not have a pheromonal signature, as sexually attractive (Billeter et al., 2009). Thus, the simplest explanation for these data is that feminized males could not sense a male-specific inhibitory pheromone, which resulted in high male–male courtship (Fig. 1). However, feminization of *ppk23*-expresssing neurons did not affect the ability of males to sense excitatory signals present in the female. Thus, when presented with a choice between a male and a female, feminized males still preferred to court females over males. In spite of the simplicity of the above model, further investigations indicated that the increased courtship toward other males by males with feminized *ppk23*-expressing cells was not purely due to the lack of sensing of an inhibitory signal. This is based on data that indicated feminized males still avoided females that were perfumed with 7-T, the main inhibitory cuticular pheromone in *D. melanogaster* (Billeter et al., 2009; Ferveur and Sureau, 1996; Lacaille et al., 2007; Wang et al., 2011) (supplementary material Fig. S4). Since the CHC profile of males is typically enriched with 7-T, our data suggest that although feminized males can sense and are repulsed by 7-T, they still find wild-type males attractive. These data showed that feminized males were actively attracted to wild-type males rather than passively defaulting to males due to the lack of an inhibitory signal, but to a lesser extent relative to their attraction to females. Furthermore, when we gave feminized and wild-type males the choice between a *D. melanogaster* male and a *D. willistoni* female, males from all genotypes (including wild-type males) preferred to court conspecific males relative to heterospecific females (Fig. 3). Together, these data suggest that males interpret the sensory input into *ppk23*-expresing cells in the context of the social environment they are exposed to. One limitation of our study is the differing strain backgrounds of our transgenic lines, and we cannot exclude that these differences may have an influence on our results. Nevertheless, our data indicate that male sexual decision-making is strongly influenced by the available mating pool. There is a possibility that the manipulations we employed in our study may have resulted in an intersex phenotype rather than full feminization. However, this would still fit our hypothesis that *ppk23*-expressing cells integrate their own sexual genetic identity with social signals to drive sexual behaviors in males. In addition, the feminization of *ppk23*-expressing neurons can lead to erroneous interpretations of the mating targets pool. These data are in further support of previous studies that showed that the social context of both males and females could affect their courtship behavior as well as the production of pheromones (Billeter et al., 2012; Kent et al., 2008; Krupp et al., 2008).

While our experimental data cannot completely exclude the possibility that feminized males were able to recognize female via non-gustatory pathways, our use of decapitated males and females as targets under red light conditions eliminated vision and the possibility that the courting males recognized sex-specific active behavioral patterns initiated by the courtship targets. Together, these data suggest that changes in the perception of contact pheromones played a role in the abnormal mating behaviors of manipulated males.

Although we have previously shown that *ppk23*-expressing cells do not overlap with sweet sensing (*Gr5a*-expressing) neurons (Lu et al., 2012), males with feminized *ppk23*-expressing neurons showed a small but significant increase in the expression of *Gr5a* receptor in their appendages. Furthermore, feminized males showed higher behavioral sensitivity to sugar stimuli. These data suggest that feminization of pheromone-sensing neurons can affect other classes of gustatory receptor neurons, possibly via indirect mechanisms. These data also further support the possible sensory crosstalk between canonical taste sensory pathways and the pheromonal input pathways as has been shown for the bitter receptors *Gr66a*, *Gr33a*, and *Gr32a* (Koganezawa et al., 2010; Lacaille et al., 2009; Miyamoto and Amrein, 2008; Moon et al., 2009; Wang et al., 2011).

Previously, we have shown that sexually-dimorphic *ppk23*-expressing neurons represent the primary *fru*-expressing GRNs in the male appendages (Lu et al., 2012). These data suggested that *ppk23*-expressing cells represent the primary subpopulation of contact pheromone-sensing GRNs. In agreement with these data, we found that feminization of all GRNs by using the pan-gustatory driver *Poxn* (Boll and Noll, 2002; Dambly-Chaudière et al., 1992) also led to male chaining behavior (supplementary material Fig. S1). However, in stark contrast to male–male courtship behaviors of *fru* mutant males (Gailey and Hall, 1989; Villella et al., 1997) and in males with feminized *ppk23*-expressing cells, males with a feminized gustatory system preferred males to females (supplementary material Fig. S1). Since *Poxn*-GAL4 is expressed in all gustatory receptor neurons including *fru*-expressing neurons in the proboscis, these data suggest that additional gustatory neurons that do not express *ppk23* are also likely to play a role in the sexual decision making process of male *Drosophila*.

Although we have previously shown that contact pheromone sensory neurons are sexually dimorphic in terms of their axonal projection patterns (Lu et al., 2012), feminization of gustatory receptor cells affected the behavior of males without an obvious gross impact on male-specific axonal patterns (Fig. 2). This outcome was surprising since previously published studies showed that manipulation of the *fru*-dependent sex determination pathway had a significant effect on axonal midline crossing of gustatory neurons in males and females (Mellert et al., 2010; Possidente and Murphey, 1989). It is possible that the lack of effect of *tra^F^* with the *ppk23-GAL4* driver is due to the late onset of *ppk23* transcription during development. *ppk23* expression begins in the late pupal stages (supplementary material Fig. S6A), and therefore *ppk23-GAL4* may not affect midline crossing in the nervous system. Rather, *ppk23* may act in the maintenance of sex-specific circuits post-developmentally. *Poxn* expression, however, begins in the embryonic stage (supplementary material Fig. S6B) and so it remains unclear why the expression of *tra^F^* with the *poxn* driver did not alter neuronal wiring patterns. Consequently, based on the current understanding of the sex-determination pathway in *Drosophila*, we expected that ectopic expression of *tra*^F^ in males would phenocopy what was reported in previous studies since *tra^F^* signaling is upstream from *fru*. Furthermore, *ppk23*-feminized males did show a significant increase in the *fru*^F^ specific transcripts in their appendages (Fig. 4A). One possible genetic explanation to the discrepancy in our findings is that we ectopically expressed *tra*^F^ in the background of wild-type *tra* locus. Therefore, it is possible that the endogenous male-specific sex-determination genetic cascade was sufficient to maintain the male-specific axonal projection pattern. Nevertheless, our data strongly support the hypothesis that certain aspects of the sexual dimorphism observed in the *ppk23*-expressing cells do not depend on their abundance in males versus females or their sexually dimorphic axonal midline crossing.

The studies we report here contribute to a better understanding of the role of the sex-determination pathway in regulating the sensory inputs used by males to make mating related decisions. Our data support a model in which *ppk23* pheromone sensing neurons represent a focal element in the sex circuit, which determines how males respond to their social environment to achieve adaptive mating decisions. Our approach indicates that by taking advantage of mosaic males in which only one class of sensory neurons is female-like in otherwise intact males would enable us to start dissecting in high detail the genetic networks that determine sexual decision making in males and females, independent of higher central neuronal functions.

## Materials and Methods

### Fruit fly strains and genetics

All fly stocks were maintained on standard cornmeal medium at 25°C under 12:12 light–dark cycle. The *ppk23 promoter-GAL4* line was described previously (Lu et al., 2012). *UAS-tra^F^* flies were from Ralph Greenspan. Unless mentioned, all other fly strains used in our studies were obtained from the Bloomington Stock Center. Non-*D. melanogaster* fruit fly species were obtained from the San Diego Species Stock Center. Specific lines used were: *D. simulans* 14011-0251.192, *D. sechellia* 14021-0248.03, *D. yakuba* 14021-0261.01, *D. erecta* 14021-0224.00, *D. ananassae* 14024-0371.16, *D. pseudoobscura* 14011-0121.104, *D. persimilis* 14011-0111.50, *D. willistoni* 14030-0811.35, *D. mojavensis* 15081-1352.23, and *D. virilis* 15010-1051.118. The used species were chosen based on whole genome availability as well as coverage of the major groups across the *Drosophila* lineage. All species were maintained on standard cornmeal medium except for *D. mojavensis*, *D. persimilis* and *D. pseudoobscura*, which were supplemented with banana, and *D. sechellia*, which was supplemented with noni fruit leather (*Morinda citrifolia*).

### Real-time quantitative RT-PCR assays

qRT-PCR was assayed as previously described (Lu et al., 2012). Briefly, fly appendages were separated by repeated vortexing of whole flies frozen in liquid nitrogen. Total RNA extraction and cDNA synthesis were performed by using Trizol and SuperScript II reverse transcriptase, respectively (Invitrogen). qPCR assays were performed on an ABI7500 machine with ABI SYBRGreen chemistry. The housekeeping gene *rp49* was used as an RNA loading control. Ct data were transformed according to the ΔΔCt method and are represented as relative values (Ben-Shahar et al., 2002). See supplementary material Table S1 for gene-specific primers used in our study.

### RT-PCR assays

Total RNA extraction and cDNA synthesis from fly appendages were performed as described above. To identify the presence of *fru* transcripts in our samples we conducted a PCR-based screen using the following forward primers: male specific *fru^M^* (GGCGACGTCACAGGATTATT), female specific *fru^F^* (TCAATCAACACTCAACCCGA), common *fru^C^* (TGGAACAATCATCCCACAAA), and a common *fru^R^* reverse primer (AGTCGGAGCGGTAGTTCAGA). PCRs were performed with Taq supermix (Lamda) in 25 µL reactions, and then separated on a 1.0% agarose gel (Fig. 4D).

### Chemical analysis of cuticular hydrocarbons (CHC)

Male flies that were 4–7 days old were kept frozen in −80°C until extraction. Parental genotypes *ppk23-GAL4* and *UAS-tra^F^* were used as controls. For CHC extraction, groups of 5 frozen flies were shaken in a glass vial with 200 µL of Hexane. 100 ng n-octadecane was added to the extracts (C-18), as an internal standard. Samples from the extract were analyzed using gas chromatography (CP 3900; Varian). Quantitative analyses of CHCs were done with a DB-1 fused silica column that was temperature-programmed from 150°C (1 min. of initial hold) at 5°C/min to 300°C. Compound quantification was done by peak integration in comparison with the internal standard. Peaks identity was verified by using a 5975 Supersonic Molecular Beam (SMB) GC-MS with cold EI (Amirav et al., 2008) (Aviv Analytical model 5975-SMB, http://www.avivanalytical.com), which provides an unambiguous molecular ion as well as pronounced ion fragments at the branching points of branched hydrocarbons. The identities of the compounds in the extracts were in agreement with previously published data (Everaerts et al., 2010).

### Histochemistry and microscopy

Immunostainings of thoracic ganglia was done as previously described (Lu et al., 2012). In short, freshly dissected brain and thoracic ganglia from flies that express a membrane tethered version of EGFP (CD8::GFP) in either *ppk23* or *Poxn* expressing neurons were fixed in 4% paraformaldehyde and washed in PBT. The specimens were co-stained with anti-GFP (Invitrogen) and the neuropil marker anti-nc82 (Developmental Studies Hybridoma Bank, University of Iowa) and mounted on slides with Slowfade Gold antifade reagent (Invitrogen) according to well-established protocols (Wu and Luo, 2006). All images were taken with a Nikon A1 confocal microscope. Shown images were constructed from optical Z-stacks and analyzed using the Nikon NIS-Elements software package.

### Courtship behavior

Courtship was assayed with 4�–7-day-old males as previously described (Ben-Shahar et al., 2010; Lu et al., 2012). In short, courtship assays were done under red light conditions unless differently stated and targets were decapitated. Courtship latency was calculated as the time from female introduction until the male showed obvious courtship behavior such as orientation coupled with wing extensions. Once courtship began, courtship index was calculated as the proportion of time a male spent in any courtship-related activity during a 10 min. period or until mating occurred. For the 7-T treatment, groups of CO_2_ anesthetized virgin 4–5-day-old females were placed in small glass vials that were coated with a thin layer of the compound. Females were then perfumed by three repeats of 20 seconds of gentle vortexing followed by a 20-second rest interval according to previously published protocols (Billeter et al., 2009). The 7-T courtship assays were performed under white light in a circular courtship arena (22 mm in diameter).

### Interspecific single-pair tests

*D. melanogaster* virgin males were collected upon eclosion and kept separately in small vials (12×75 mm). Female virgin flies of all species were collected upon eclosion and kept in groups of up to 10 flies from a single-species. All vials contained standard cornmeal medium. Flies were aged 4–7 days under constant conditions of 25°C and a 12:12 light–dark cycle before behavioral experiments to ensure reproductive maturation. Interspecific no-choice tests were then carried out in behavioral chambers as previously described (Lu et al., 2012).

### Chaining behavior

Male chaining was assayed with eight male in a 22 mm diameter circular arena as previously described (Lu et al., 2012). Chaining index was calculated as the proportion of time in which at least three males showed chaining courtship to each other during a 10 min. observation.

### Choice behavior

Choice was assayed by introducing a single focal male and two decapitated targets. Flies were videotaped and analyzed for the duration of time the focal male spent courting each of the two targets. The courtship choice index was calculated [(duration of courtship of target A – duration of courtship of target B)/total courtship time]. Courtship time was measured from the moment the male started courting one of the targets. Total assay time was kept at 10 min.

### Proboscis extension reflex

Proboscis extension reflex (PER) assays were as previously described (Lu et al., 2012). In short, 1-day-old flies were starved for approximately 24 hours, then immobilized by chilling on ice and mounted ventral-side-up using myristic acid. Flies were allowed to recover for two hours under humid conditions. Flies were satiated with water prior to the PER training. Flies were tested by introducing a drop of the test solution to a foreleg. Only full PER responses were recorded as positive. Each fly was exposed three times to the same stimulus in each concentration with water application between each trial. ‘Responders’ were classified as such if they responded to at least 2 out of 3 trials. The responding index represents the sum of all positive responses of an individual animal to a specific sequence of tarsal stimuli.

### Statistical procedures

All statistical tests were performed using the R statistical package. Data were tested for normality by using the Shapiro–Wilk test. Two-sample t-tests and one-way ANOVA tests were used for parametric statistics and the two-samples Wilcoxon test and Kruskal–Wallis rank sum test were used for non-parametric tests. Chi-square tests were used for frequency-based data.

## Supplementary Material

Supplementary Material
