# Supplementary material for: Feminization of pheromone-sensing neurons affects mating decisions in Drosophila males
Source: Biol Open. 2014 Jan 17;3(2):152–60. doi: 10.1242/bio.20147369 (PMC3925318; doi:10.1242/bio.20147369)
Supplement: Supplementary Material [file supp_3_2_152__index.html]

Feminization of pheromone-sensing neurons affects mating decisions in Drosophila males — Feminization of pheromone-sensing neurons affects mating decisions in Drosophila males — Supplementary Material 

# Feminization of pheromone-sensing neurons affects mating decisions in *Drosophila* males

## bio.20147369 Supplementary Material

**Files in this Data Supplement:**

- Supplementary Material - Beika Lu et al. doi: 10.1242/bio.20147369
