# Supplementary material for: Feminization of pheromone-sensing neurons affects mating decisions in Drosophila males
Source: Biol Open. 2014 Jan 17;3(2):152–60. doi: 10.1242/bio.20147369 (PMC3925318; doi:10.1242/bio.20147369)
Supplement: Supplementary Material [file supp_bio.20147369_bio.20147369-s1.pdf]

Biology Open

Supplementary Material

Beika Lu et al. doi: 10.1242/bio.20147369

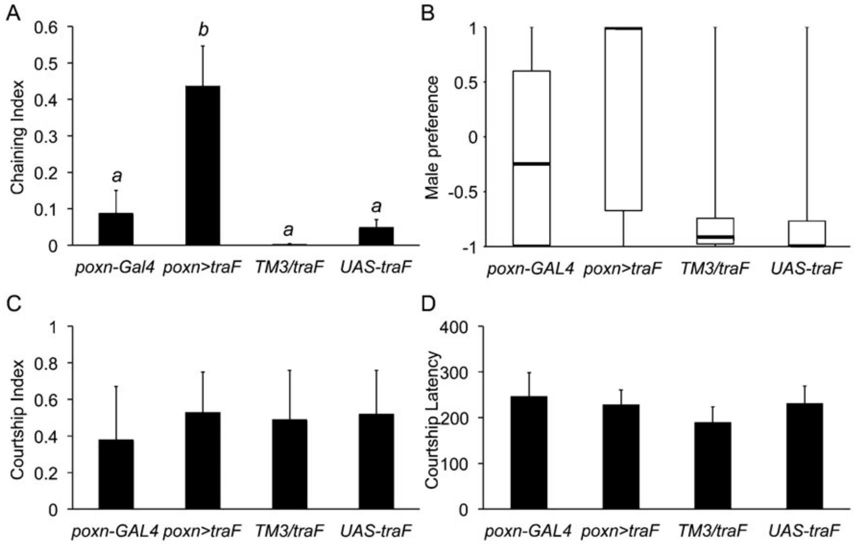

Fig. S1. Male flies with feminized *Poxn*-expressing cells show increased sexual attraction to male targets. (A) Males with a feminized gustatory system (*poxn*>*tra*^{F}) show a robust male chaining behavior relative to parental males and sibling controls (TM3/*tra*^{F}) (ANOVA, n=7–11 groups, p<0.001***). (B) Feminized males show increased male preference relative to controls (Kruskal–Wallis rank sum test, n=22–25, p<0.001***). Boxplots show the distribution of choice behaviors (1, male; −1 female). (C,D) Males with feminized gustatory system show normal courtship behavior towards wild-type females measured by courtship latency and index (Latency: Kruskal–Wallis rank sum test, n=22–25, p=0.72; Index: Kruskal–Wallis rank sum test, n=22–25, p=0.33).

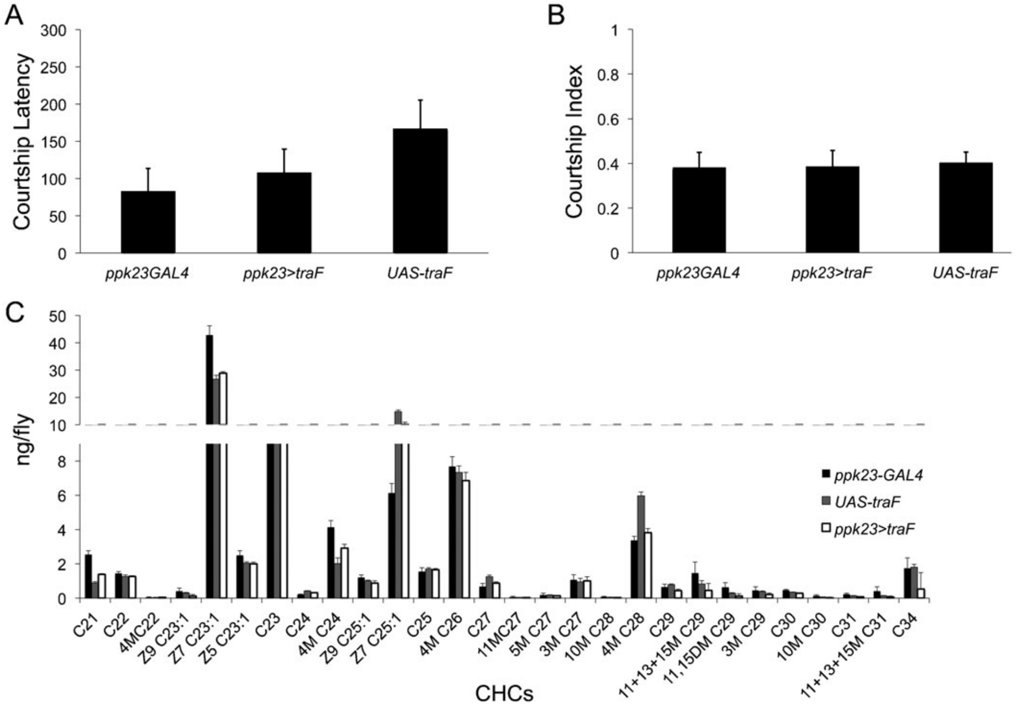

Fig. S2. Males with feminized *ppk23*-expressing cells are not sexually attractive to wild-type males. (A) Courtship latency (ANOVA, n=19–25, p=0.18). (B) Courtship index (ANOVA, n=19–25, p=0.96). (C) The cuticular pheromone profiles of males with feminized *ppk23*-expressing cells are not different from wild type (ANOVA, n=10 samples per genotype).

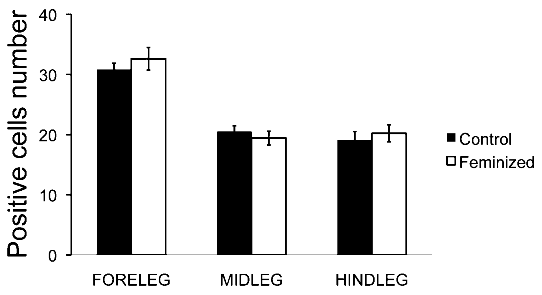

Fig. S3. Feminization of *ppk23*-expressing neurons does not affect their abundance in male legs (ANOVA, *n*=10 per leg for each genotype). Genotypes were as in Fig. 4. Cells were counted blindly under a fluorescent microscope.

A

Genotype	*ppk23*-GAL4	*ppk23>tra^F^*	*UAS-tra^F^*
7-T	35.71%	43.75%	26.67%
Hexane	10%	11.11%	9.09%

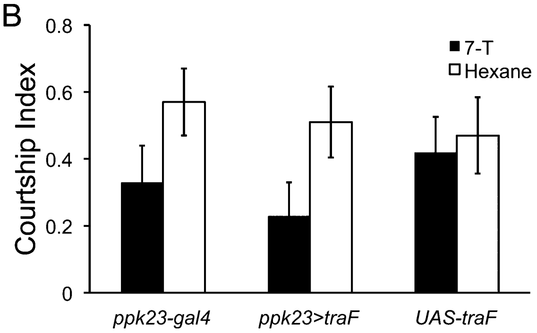

Fig. S4. Males with feminized *ppk23*-expressing cells can detect and respond to the male inhibitory pheromone, 7-tricosene (7-T). (A) Similar proportions of feminized and wild-type males were inhibited from any courting of 7-T perfumed decoys in a 10 min. observation period. (B) Males with feminized *ppk23*-expressing cells exhibited reduced courtship index to 7-T perfumed decoys that was not different from parental control lines.

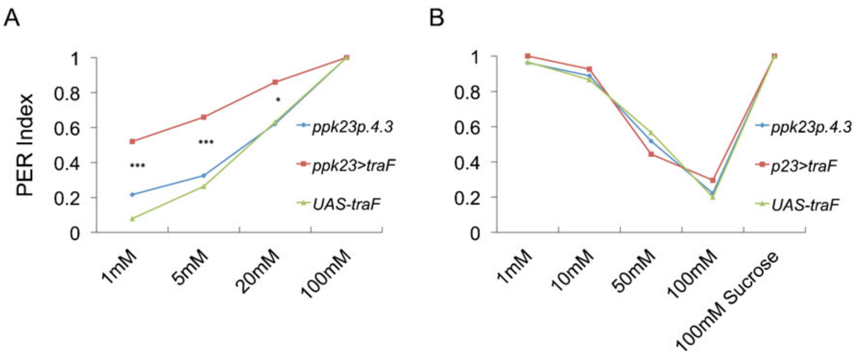

Fig. S5. Feminization of *ppk23*-expressing neurons affects the response threshold to sugar but not bitter stimuli. (A) Induction of the proboscis extension reflex in response to increasing concentrations of sucrose. Feminized males displayed lower PER threshold relative to parental genotypes (Chi-squared test; ***$p<0.001$; *$p<0.05$). (B) Feminized males displayed normal response profiles to the bitter compound caffeine (Chi-squared test; NS).

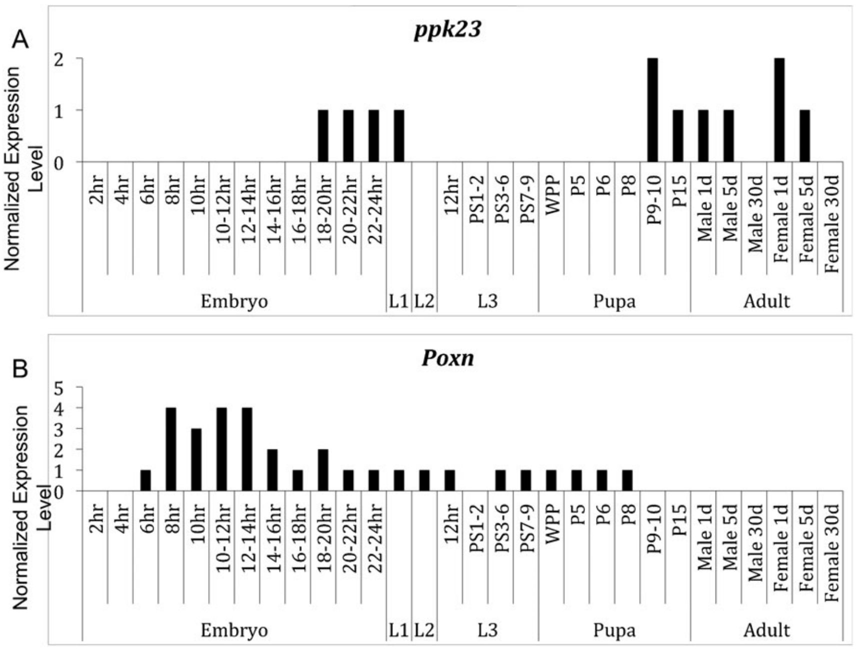

Fig. S6. *ppk23* transcription begins late in the pupal stage while *Poxn* is expressed earlier than *ppk23* in the embryonic stage. modENCODE RNAseq expression data for (A) *ppk23* and (B) *poxn* in *D. melanogaster*.

Table S1. qPCR primers.

Gene	Forward primer	Reverse primer
fruF	GTTCGGGTTGAGTGTTGATTG	AACTGTGAGAATTCGAGGACG
Gr5a	AGTTCAGGGCAAGATGTGG	TCGTGTAGGAAGTTCTTTCGC
Gr32a	TCTTTGTCCAGATTACCGGC	GAATTTGGGATGTGGCATTGG
Gr33a	TGGGATTGTTTGCTCTGGAC	TTATGACATTAGCCCCTCGTTG
Gr39a	AGATGCTGACAAAAGTACCCC	GCGGTTAAAATGGGCTTCTG
Gr66a	GGAACAAGCCAATCACGAAAG	GAGAAATCCCGTAATGGCATG
Gr68a	CAGAAGTGGCTGATACCATAGG	GCTTGCTCTTGCCGTAAATTC
ppk23	ACCGACTTCCACAACCA	GGGTATACATTTGGGCCTG
ppk25	CATATCGCAAGTGTCGCT	CACTCAAGTCGGCAAAGA
cyp6a8	AATGGTGTACAGTCGCAGAG	TTCAAGATAAGGTTCGGGCTG
cyp6a9	TGTCCAGGAACCTCATAGTCC	GTTATTGGAAAGTACAACGGAGAG
cyp6a17	GGAGCAGGTTGTGATGGAAA	CATAGTGAATGCCCAAAGCTG
cyp6a19	TGCAATCAGGATGTTCAGGAC	ACCACTGGATGTTTCCTTAGC
cyp6a20	GGGAAATTGGATTACGACAGC	TGGTGTGCTGGTAGTGTTG
cyp6a21	TGTACAATCGAAGGGTTTCCG	AATCAGGACATTCAGAACCGG
cyp6a22	CGGTACATGGAACTGGTCATAG	CGGCTCGATGTAGAAATGACG
cyp6a23	ATTCAGGATCAACTTAGGGCG	AGAACTGGATACTTGCGAAGG
cyp6d2	CCAGGTACGGGATAATCTTCG	AAGCCCAAGGATAGGTTCAC
